# Pilonidal sinus involving the breast in a man: A case report and literature review

**DOI:** 10.1097/MD.0000000000025166

**Published:** 2021-03-26

**Authors:** Rong Shi, Peng Liu, Xuan-Fen Zhang

**Affiliations:** a Department of Mammary Glands, Gansu Provincial Hospital; b Lanzhou University Second Hospital, Lanzhou City, Gansu Province; c Department of General Surgery, The 949 Hospital of PLA, City ALeTai City, Xinjiang Province; d Department of Plastic Surgery, Lanzhou University Second Hospital, Lanzhou City, Gansu Province, PR China.

**Keywords:** breast, male, pilonidal sinus disease

## Abstract

**Rationale:**

Pilonidal sinus disease (PSD) involving the breast is extremely rare and has not been described in man.

**Patient concerns:**

This current case report presents a case of a pilonidal cyst in a 46-year-old man which was surgically treated. He had intermittent pain in his left breast for 2 months and came for local rupture and discharge for 1 week.

**Diagnosis:**

The initial diagnosis is male mastitis, on the basis of the histological features of H&E-stained specimens and immunohistochemistry of the resected lump, this case was diagnosed as PSD.

**Interventions:**

The patient underwent “enlarged resection of the left breast lesion” under local anesthesia.

**Outcomes:**

The patient's surgical area healed well, without any signs of recurrence.

**Conclusion:**

PSD involving the breast is extremely rare in man, with no typically clinical manifestations, and could be easily ignored. This disease requires great attentions from clinicians.

## 1 Introduction

Pilonidal sinus disease (PSD) is a subcutaneous infection characterized by repeated rupture to form the sinus, its clinical manifestations are abscess or chronic persistent secretion. PSD occurs mostly in the intergluteal cleft or sacrococcygeal while rarely in axilla, umbilicus, submental area, nose, ear, finger and toe web spaces, groin, suprapubic area, clitoris, prepuce, penis, occiput, or female's mammary glands breasts.^[1–9]^ To our knowledge, there is no reported case of PSD occurs in male mammary gland. In order to improve the cognition of PSD and make the correct diagnosis and treatment, We analyze the clinical data, related images, and some pathological features of a male breast PSD patient and reviews pertinent literature of PSD as well.

*The patient of the case reported in this article has signed the informed consent for publication.*

## 2 Case report

### 2.1 Clinical data

The patient is a 46-year-old male, Han nationality, native to Country Huining, City Dingxi, Province Gansu, working as a coal miner, body mass index =23.7 kg/m^2^. Through his own description, we got a message that he had intermittent pain in his left breast for 2 months and local rupture and discharge for 1 week. He used to have unstable angina and gallstones, smoked an average pack of cigarettes a day for 20 years, and had no history of breast trauma.

The examination revealed that his left breast was larger than the right breast. There is a rupture with a diameter of about 2 mm under the left papilla and a small amount of yellowish purulent secretion in the rupture. The soft in texture, poor mobility, unclear boundary, and about 15 mm× 5 mm mass in size is touched, pain is not obvious under press. we initially diagnosed the patient with male mastitis and performed “enlarged resection of the left breast lesion” under local anesthesia.

### 2.2 Ultrasonic examination data

**Figure 1:**
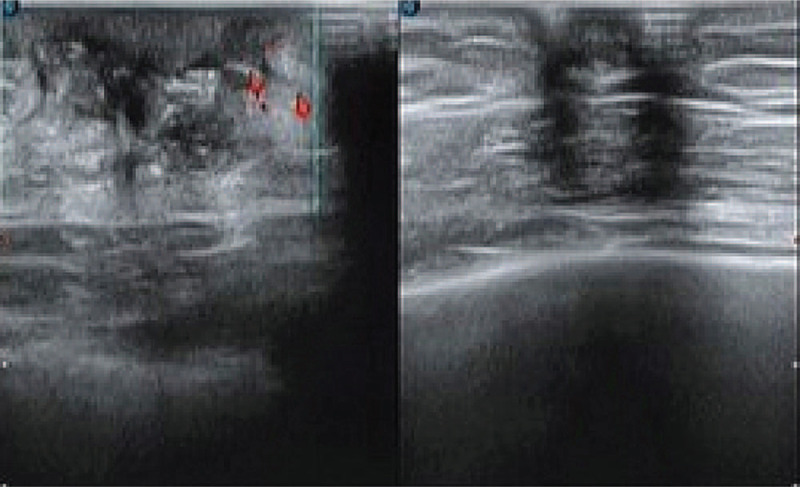
The hypoechoic region of the left breast is “V” distribution, the boundary is clear, the shape is irregular, there is no obvious blood flow signal in the CDFI.

Breast ultrasound measurements showed that 21 mm× 11 mm of uneven echo could be seen under the skin of the left breast, strong echo of gas could be seen inside, flow could be seen in the extrusion probe, and blood flow signal could be seen around the color doppler flow imaging: hypoechoic left mammary gland, consider abscess (Fig. 1).

### 2.3 Intraoperative condition

**Figure 2:**
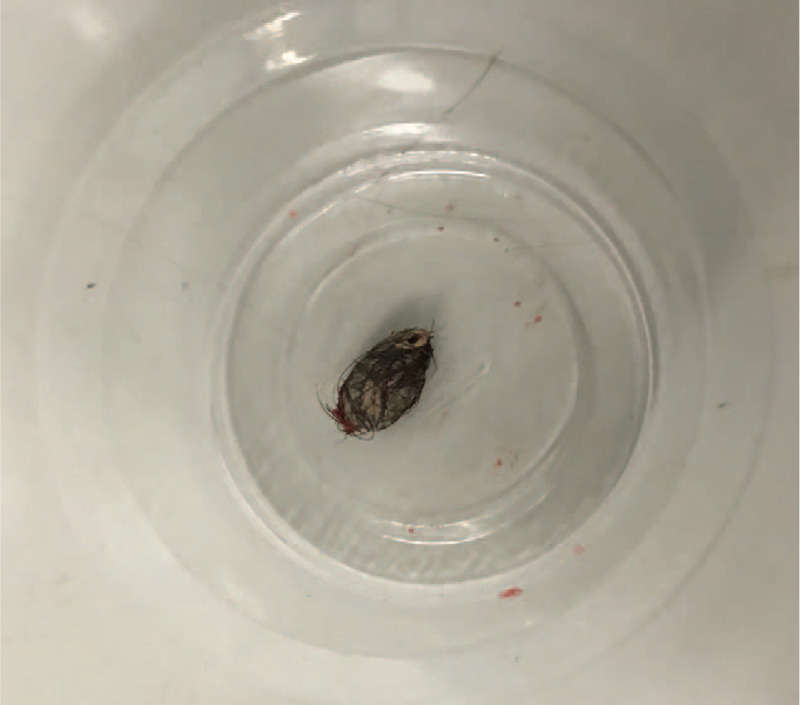
The mass is about 15 mm× 5 mm in size, with clear boundaries and covered hair.

During the operation, we can see a mass about 15 mm× 5 mm size during the operation, with clear boundaries and covered hair (Fig. 2).

### 2.4 Histopathological examination

**Figure 3:**
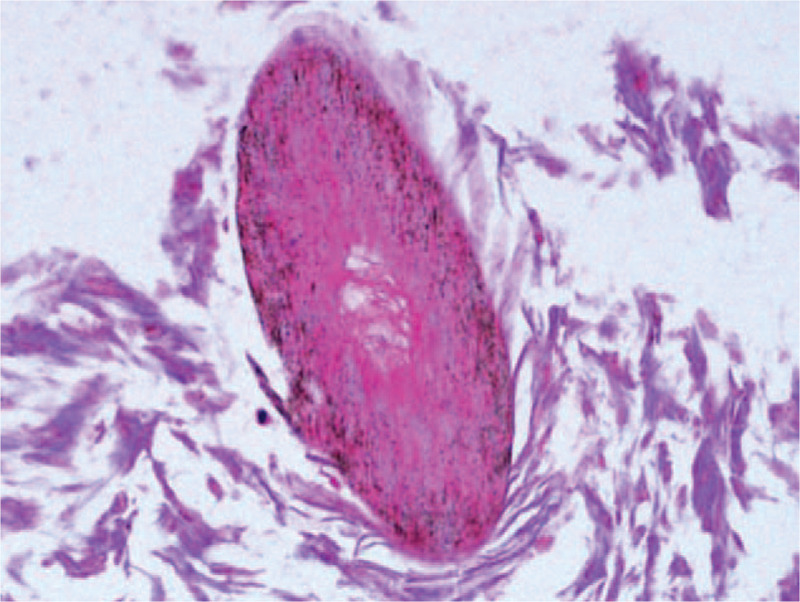
Pathologic examination showed hair shaft material in a duct with associated chronic inflammation (HE, ×400).

Left breast lesions gray-yellow nonplastic tissue piece, size 12 mm× 10 mm× 6 mm, attached hair tissue, are keratides and hair, it is very likely to be pilonidal sinus (PS). (Fig. 3, HE, ×400)

### 2.5 Postoperation and follow-up

**Figure 4:**
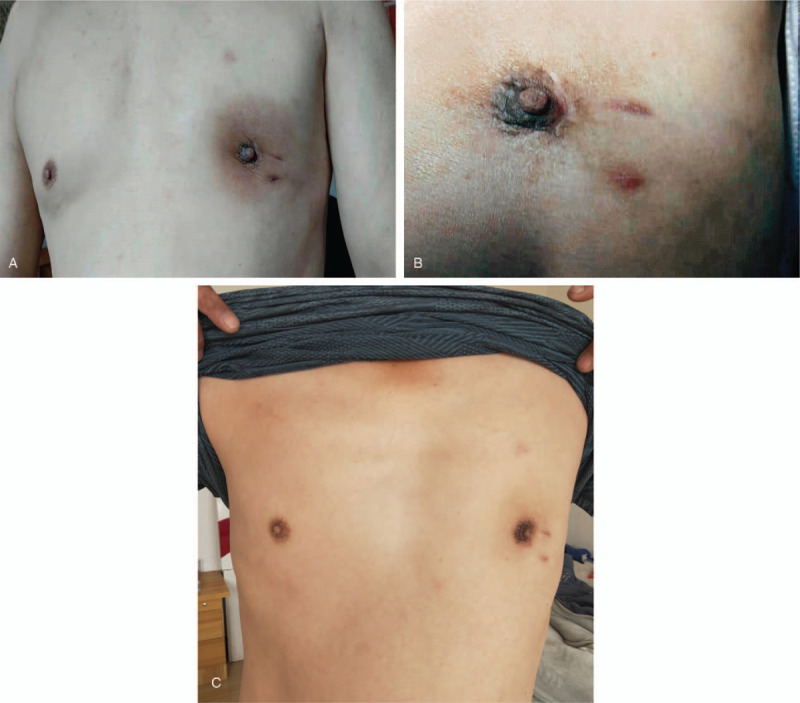
The healing condition of the wounds. A. One month after the surgery, the operation area recovered, with pigmentation around the wound. B. Two months after the surgery, the operation area recovered well and the scar can be seen. C: Six months after the surgery, the operation area recovered well.

The patient had recovered well after “enlarged resection of the left breast lesion” under local anesthesia in our hospital on October 18th, 2019. One month, 2 months, and 6 months after the operation, we followed up the patient's recovery by telephone, respectively. We knew that the patient's surgical area healed well, no recurrence and we will follow up for a long time (Fig. 4A–C).

## 3 Discussion

The disease was first described in 1833 Herbert Mayo, The term “pilonidal” means “nest of hair,” however, it was not until 1880 that Hodges officially named the PS in Latin (hairy) nidus (nest).^[10–11]^ For a time it was known as “Jeep disease” due to high incidence in soldiers during World War II, where it affected several thousand combat troops.^[12]^ The incidence in Europe and the United States is nearly 26/100,000,^[13]^ and the incidence in Asian is relatively low. As a result of the relatively low incidence in Asian countries, there are currently no epidemiological data on the disease. People aged 10 to 40 are more likely to develop the disease, and men are 3 to 4 times more likely than women. Common risk factors of pilonidal disease include: male, hairy physique, obesity, sacrococcygeal skin trauma, poor personal hygiene, sedentary habits, etc. During the study, Shareef and colleagues observed that female patients with intermammary PSD had the habit of wearing tight bras, which increased intermammary regional pressure, enhanced hair penetration into the skin, and increased incidence of PS infections.^[14]^ Another literature showed that the disease is of professional relevance and is more common among female hairdressers, wool pedicure, and dog beauticians.^[16–19]^ American Society of Colon and Rectal Surgeons advocate hair depilation to decrease the risk of recurrence in patients with PS.^[20]^ Although conventional laser hair removal after surgery can also reduce the risk of recurrence, there is no evidence to recommend this technique routinely. Researcher also found that recurrent hair removal could cause initiating trauma that could trigger the development of PS.^[21]^

PSD is now understood to be an acquired condition, It usually presents as a cyst, abscess, sinus tract, singly or in clusters. PSD is a common, perianal, recurring, inflammatory condition caused by hair penetrating to the outer layer of skin,^[6]^ the incidence of PSD is 0.07% and account of 15% of perianal diseases,^[22]^ it rarely occurs in other parts of the body. Shareef observed 12 patients with intermammary PSD, but all cases were female.^[15]^ The current study confirmed that being hairy is not necessary for the formation of PSD as all cases had not had hairs at the site of the disease.^[9]^ In our report, we did not find that the patient had the risk factors mentioned neither, such as obesity, hairy, sedentary, and other risk factors. This is also consistent with the results observed by Cubukçu's observation that the body mass index is no significant difference between patients with PSD and patients with other diseases.^[23]^

In the current German guidelines, excision and open therapy are still designated as standard techniques. Nevertheless, in the English version of the same guide, the reviewers refused to use the word “standard” to describe it because they thought it conflicted too much with the current state of evidence.^[24]^ When it comes to the treatment of PSD, the current treatment methods are mainly divided into nonsurgical treatment and surgical treatment. Nonsurgical treatment includes:

1. Anti-inflammatory treatment: it as an adjunct to surgical treatment;
2. Sclerotherapy: inject of 80% phenol solution or anhydrous alcohol into the sinus tract;
3. Lu used traditional Chinese medicine thread pulling and cotton pad compression therapy to treat PSD and achieved satisfactory results.^[25]^

Surgical therapy is the most fundamental and thorough cure after the diagnosis of PSD. To be given anti-inflammatory therapy when with infection; After abscess forms, incision and drainage, excision of the lesion and primary closure (off-midline wound closure can be considered), open healing, rotation flaps, and cleft-lift can be performed. There is still lack of clear evidence to specifically guide the treatment of recurrent PSD. At the rate of wound healing, the primary healing of PSD surgery is faster than open healing. But the risk of relapse is high. Off-midline closure should become standard management for PSD when closure is the desired surgical option.^[26]^

PSD should be distinguished from skin-derived infectious diseases (furuncles, carbuncle, cellulitis, hidradenitis suppurativa), sebaceous gland cyst, teratoma, plasma cell mastitis, and mastitis. PSD is easy to be misdiagnosis and mistreatment. Long-term recurrent attacks may cause infections, abscesses, and the risk of carcinogenesis, the most common of which is squamous cell carcinoma.^[27]^ Since the PSD occurs in the working age, its economic impact on patients can not be ignored. At the same time, it can cause physical and psychological trauma to patients. In clinic, it is difficult to differentiate the pathological features by observing the pathological features, and the histopathological examination and immunohistochemistry are needed to confirm the diagnosis.

## Author contributions

**Conceptualization:** Rong Shi, Peng Liu.

**Data curation:** Peng Liu.

**Writing – review & editing:** Rong Shi, Xuan-fen Zhang.
